# Current Knowledge on the Different Characteristics of Back Pain in Adults with and without Scoliosis: A Systematic Review

**DOI:** 10.3390/jcm12165182

**Published:** 2023-08-09

**Authors:** Fabio Zaina, Rosemary Marchese, Sabrina Donzelli, Claudio Cordani, Carmelo Pulici, Jeb McAviney, Stefano Negrini

**Affiliations:** 1 ISICO (Italian Scientific Spine Institute), 20141 Milan, Italy; sabrina.donzelli@isico.it (S.D.); carmelo.pulici@isico.it (C.P.); 2 ScoliCare, Sydney 2217, Australia; rosemary.marchese@scolicare.com (R.M.); jeb@scolicare.com (J.M.); 3 Department of Biomedical, Surgical and Dental Sciences, University “La Statale”, 20122 Milan, Italy; claudio.cordani@unimi.it (C.C.); stefano.negrini@unimi.it (S.N.); 4 IRCCS Istituto Ortopedico Galeazzi, 20157 Milan, Italy

**Keywords:** scoliosis, low back pain, back pain, disability, lumbar spine

## Abstract

Patients with scoliosis have a high prevalence of back pain (BP). It is possible that scoliosis patients present with specific features when experiencing back or leg pain pathology. The aim of this systematic review is to report the signs, symptoms and associated features of BP in patients with scoliosis compared to adults without scoliosis during adulthood. From inception to 15 May 2023, we searched the following databases: PubMed, EMBASE, the Cumulative Index to Nursing and Allied Health Literature (CINAHL), and Scopus. We found 10,452 titles, selected 25 papers for full-text evaluation and included 8 in the study. We found that scoliosis presents with asymmetrical pain, most often at the curve’s apex, eventually radiating to one leg. Radiating symptoms are usually localised on the front side of the thigh (cruralgia) in scoliosis, while sciatica is more frequent in non-scoliosis subjects. These radiating symptoms relate to rotational olisthesis. The type and localization of the curve have an impact, with lumbar and thoracolumbar curves being more painful than thoracic. Pain in adults with scoliosis presents specific features: asymmetrical localization and cruralgia. These were the most specific features. It remains unclear whether pain intensity and duration can differentiate scoliosis and non-scoliosis-related pain in adults.

## 1. Introduction

Idiopathic scoliosis is a three-dimensional spine and trunk deformity of unknown origin [1]. There are several classifications based on the location and size of the curves and according to the age of diagnosis [1]. Usually, idiopathic scoliosis becomes evident during adolescence (AIS), which is the riskiest period for worsening due to rapid growth. Infantile and juvenile scoliosis are less common but, in many cases, show a more unfavourable prognosis [2]. Occasionally, idiopathic scoliosis is diagnosed later, during adulthood, while primary (de novo) degenerative scoliosis refers to a structural curve that develops after skeletal maturity in a previously normal spine [3]. It is also a fairly frequent condition, especially in females, with a prevalence of up to 37.6% in people older than 60 years [4].

Despite etiological differences, the clinical impact on Quality of Life (QoL) of idiopathic and degenerative scoliosis during adulthood can be similar. Studies have shown that patients with scoliosis have a higher prevalence of back pain (BP) and experience a more severe and longer duration of pain than controls without scoliosis [5]. Pain can eventually radiate distally to one or both legs. Features that distinguish BP related to scoliosis, as opposed to other potential causes of BP, have yet to be identified. Pain in scoliosis patients seems to have specific features, including increasing with prolonged standing while reducing when lying down [5]. Also, the localization of pain seems different in patients with scoliosis, with the pain being more asymmetric and principally at the apex of the curve, on either the side of the prominence or the concavity and frequently radiating to one of the inferior limbs [6]. Most of the time, the pain is localised in the lumbar spine, which is subjected to faster degeneration effects; however, in some cases, pain is localised in the thoracolumbar or in the thoracic spine in the prominent area where the biomechanics play a major role [7]. This is why authors sometimes refer to BP and other times to low back pain (LBP).

According to current knowledge, AIS should reach the threshold of 30° to be significant in adulthood [8,9], while degenerative scoliosis can be painful even at lower degrees [4]. Unfortunately, in everyday clinical practice, it is not always possible to differentiate between the two forms. We can diagnose scoliosis as indeed being degenerative only if it is lumbar/thoracolumbar and we have a previous radiograph showing a straight spine. However, degenerative phenomena may also affect idiopathic scoliosis during adulthood. 

According to some estimates, we can expect that by 2050, the proportion of the world’s population aged greater than 60 years will nearly double [10]. This event will increase the number of patients with scoliosis presenting to doctors with BP [11]. Therefore, there is a need to better identify the clinical and associated features of BP in adult patients living with scoliosis to distinguish whether scoliosis is the underlying cause of BP. Understanding the features of BP in this group of patients would have clinically relevant outcomes related to the treatment and prevention of pain. 

The primary aim of this systematic review is to report and characterise the signs, symptoms and associated features of pain (e.g., localization, intensity, duration, modifying factors) in patients with idiopathic or degenerative scoliosis during adulthood compared to adults without scoliosis. The hypothesis is that scoliosis patients present specific features when experiencing back or leg pain connected to the peculiarities of the structural changes of their spine. 

The secondary aim is to differentiate LBP and leg pain features between idiopathic and degenerative scoliosis.

## 2. Materials and Methods

### 2.1. Design

We developed this systematic review based on the MOOSE Reporting Guidelines for Meta-analyses of Observational Studies [12]. We registered the protocol on PROSPERO (CRD42023364455).

### 2.2. Selection Criteria

#### 2.2.1. Type of Study

We included original peer-reviewed primary research articles that were considered a control group. We considered studies in any language, and we obtained translations where needed. We excluded secondary research (review articles), case reports and studies that did not meet the inclusion criteria.

#### 2.2.2. Population

We included adults with scoliosis and BP or LBP. The definition of scoliosis in adults included adults diagnosed with idiopathic scoliosis as an infant, juvenile or adolescent or those diagnosed with scoliosis during adulthood (idiopathic or degenerative). We included these different types of scoliosis because, in clinical practice, it can sometimes be difficult to be certain whether they are degenerative, idiopathic or even both. Moreover, most of the published studies presented a mixed population. Finally, despite some differences, we can expect similar complaints. We excluded studies if the scoliosis was not idiopathic or degenerative, such as neuromuscular, congenital and other secondary scoliosis. We also excluded studies if the patients underwent surgical management for their scoliosis. We included studies of patients treated during adulthood, provided they did not receive any treatments in the last six months, and considered only the baseline information (i.e., before any treatment is applied). 

#### 2.2.3. Search Strategy

From inception to 15 May 2023, we conducted a literature search in the following databases: PubMed (via https://pubmed.ncbi.nlm.nih.gov/ accessed on 15 May 2023), CINHAL (via EBSCOhost), EMBASE (via Embase.com) and Scopus. In addition, we searched the reference lists of the included studies for other possible studies. We contacted the authors for studies in which the full text was unavailable. We first developed the search strategy for PubMed and adapted it to the other databases.

Search strings were composed of search terms defining the “scoliosis” OR “spinal deformities” AND “low back pain”, “spinal pain” OR “pain”.

**Figure 1:**
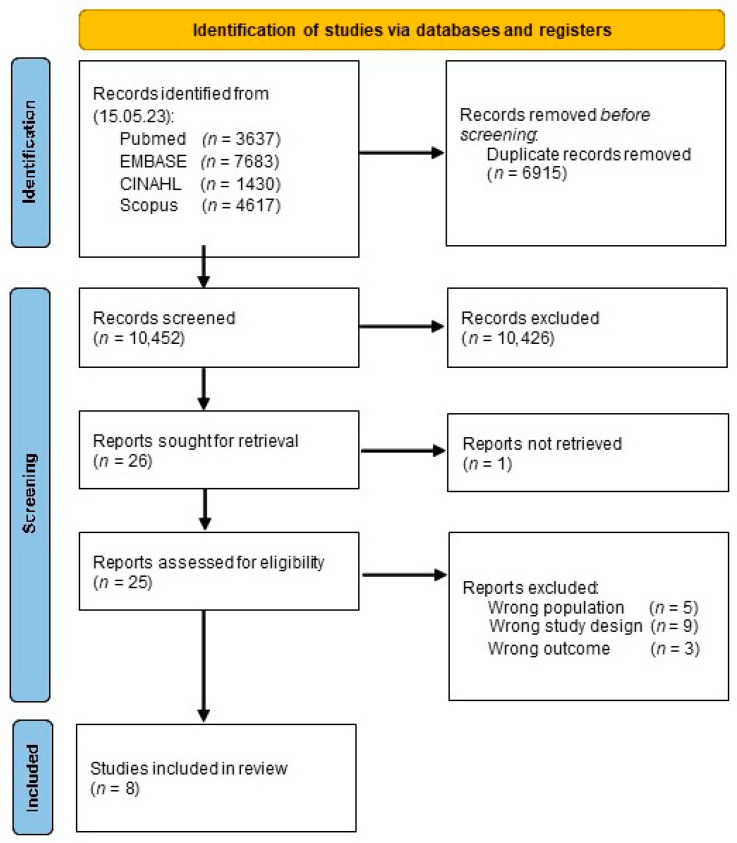
Study flow chart.

The complete search strategies for each database are available in Appendix A. We imported the search results into the bibliographic management online software Rayyan (https://www.rayyan.ai accessed on 15 May 2023) after we discarded duplicates on EndNote X9. We reported the results of the search as per the MOOSE flow diagram (Figure 1).

#### 2.2.4. Outcome Measures

The outcomes of interest are the signs, symptoms and associated features of BP and LBP in adults with and without scoliosis. Pain-related outcomes may include but are not limited to intensity, duration, type, location (back or distal), onset and triggering factors/positions, relieving factors/positions, and time-related behaviour. Associated features may include but are not limited to patient demographics (gender, age, occupation), number of pregnancies, family history of scoliosis and pain, Cobb angle, number of curves, types of curve and X-ray features, e.g., osteoporosis, rotational olisthesis.

#### 2.2.5. Study Screening

Two reviewers (CP, FZ) independently screened the titles and abstracts retrieved by the search strategy and assessed the full-text articles for their potential inclusion. Disagreements were resolved through discussion with another author (RM) to reach a consensus. We managed these phases by using the Rayyan software.

#### 2.2.6. Data Extraction

Two reviewers (CP, FZ) independently extracted the general characteristics (first author, publication year, study design, study setting, sample size, participant characteristics) and outcome data into an Excel form. We solved any differences in opinion about the study characteristics with a third review author (RM).

### 2.3. Quality Assessment 

Two reviewers (CC, SD) independently assessed the studies’ quality. We solved any differences in opinion about the methodological quality with a third review author (SN). We used the JBI checklist, as appropriate.

### 2.4. Evidence Synthesis and Statistical Analysis

We tabulated the characteristics of the included studies for comparison. We intended to assess for heterogeneity (e.g., visually, using I^2^ or the χ^2^ test) and, if possible, include a prevalence meta-analysis with weighted proportions. However, due to the small number and some limitations of the included studies, we performed a narrative synthesis with frequencies because the meta-analysis was not applicable.

## 3. Results

### 3.1. Study Selection 

After removing the duplicates from the different databases, we found 10,452 titles (Figure 1). After the title screening, we selected 25 papers for a full-text evaluation and included 8 in the study. (Figure 1, Table 1).

The reasons for exclusion were no study design of interest (nine papers), no population of interest (five papers) and no outcome of interest (three papers).

Three studies were prospective controlled [6,13,17], three were retrospective [5,7,15], and two were cross-sectional [14,16]. One of the cross-sectional studies was a congress abstract [16]. Two studies were from France, two were from Canada, and the others were from the USA, The Netherlands, China and Italy.

The total number of scoliosis patients was 727, and the controls were 1590.

Three studies included a larger number of adults with scoliosis and healthy controls but were included because they presented data for the subgroup of patients with BP [5,7,17].

Five studies focused on LBP [6,13,14,15,16], while the other three reported on BP, including both thoracic and lumbar or without giving details on the location [5,7,17]. 

### 3.2. Critical Appraisal

Following the JBI checklist, in the cross-sectional studies [14,16], the major limitations were the absence of strategies to identify and manage confounding factors. Moreover, in one study [16], the selection criteria and statistical analysis were not completely clear. Regarding the longitudinal studies [5,6,7,13,15,17], the main methodological limitations were associated with the absence of the confounding factors’ identification and the strategies for managing them, as well as the application of strategies to address incomplete follow-up visits. Table 2 provides the results of the critical appraisal performed on the studies included in the present review.

### 3.3. Main Findings

The description of symptoms (localization, intensity, disability and functional status) varied among the different papers. Five studies reported pain localization, five studies reported pain severity and/or disability in adults with scoliosis compared to non-scoliosis subjects and two papers reported on the factors influencing pain (Table 3).

### 3.4. Pain Localization

Five papers reported pain localization [6,7,13,14,16]. Two papers reported on a similar population of older people, and therefore we pooled their data [6,13]. One was about younger subjects [14]. One congress abstract reported sciatica prevalence [16].

In two studies, adults with scoliosis and LBP experienced more frequent radiating pain and cruralgia (defined as compressive nerve root irritation of L3–L4 [18]) than the control group of LBP patients without scoliosis (48 vs. 37.5% and 20% vs. 6.7%, respectively), and sciatica was more frequent in patients without scoliosis (26% vs. 44%) [6,13]. A congress abstract reported similar significant findings (27 vs. 47%) [16]. Cruralgia was associated with rotatory dislocation (olisthesis) [6,13].

In one paper reporting on the younger adult population, all the scoliosis patients experienced unilateral lumbar pain (78% of the time on the convex side), while 83.7% of patients without scoliosis experienced midline or symmetrical lumbar pain [14].

Considering the back area, the most common localization of pain was over the major deformity in scoliosis. In a double major curve, the pain was frequently at the distal curve, while in thoracic curves, the pain was at the distal junctional level [7].

### 3.5. Pain Intensity and Disability

Five studies described pain intensity and disability [5,12,14,16,18]. One study reported pain intensity and frequency at 50 years of follow-up [17]. The authors reported that pain intensity and duration were similar between scoliosis and non-scoliosis adult patients with BP [17]. They also created a more complete pain composite, summing the pain intensity and duration. Also, this parameter showed similar trends in both groups [17].

One congress abstract reported similar findings for pain and disability in scoliosis and non-scoliosis adults with LBP [16]. The numerical rating scale (NRS) values were 5.9 ± 1.8 for scoliosis patients versus 5.1 ± 1.2 for the controls, while the Oswestry Disability Index (ODI) values were 33.9 ± 17.6% versus 32.6 ± 18.8% [16].

A retrospective study included subjects with chronic BP who failed a primary care conservative treatment approach and were referred to a combined physical and psychological program. The authors found no differences at the baseline for pain intensity (58.4 ± 19.1 vs. 60.4 ± 19.1 for NRS), functional status (39.5 ± 12.0 vs. 40.2 ± 12.1 for ODI), or pain duration (15.5 vs. 13.6 years) [15].

On the contrary, two retrospective studies reported more frequent pain in scoliosis patients [5,7]. One study found that current BP and prevalence of BP over the last year were higher for scoliosis than non-scoliosis adults, without any impact of curve entity [5]. In scoliosis patients, the pain was more continuous and chronic [5]. The other study found that adults with scoliosis had more severe, constant or frequent pain, while non-scoliosis patients referred more occasional or recurrent pain [7].

### 3.6. Factors Influencing Pain

Two papers reported data on the factors influencing pain [5,7]. One paper reported details from the Roland Morris Scale (RM), the ODI and McGill Pain Questionnaire [5]. Compared to non-scoliosis BP patients, adults with scoliosis and BP showed a more frequent need to change position, with limitations in standing and sitting for a long time [4]. Patients with curves larger than 40° also showed limitations in walking, and those with curves between 20° and 40° had limitations in lifting and travelling [5]. Issues related to social activity, personal care and the need for pain control were similar among the two groups [4]. One retrospective study reported that major lumbar, thoracolumbar and lumbosacral curves were the most painful, while major thoracic was the least painful [7]. 

## 4. Discussion

There is evidence that adults with scoliosis frequently report pain issues. In clinical practice, it is sometimes difficult to understand whether the pain relates to the spinal deformity or is nonspecific [19]. BP is so common that there are cases in which it affects someone with scoliosis just by chance. To help clinicians, we designed this systematic review to report the available information on the topic. Only a few studies compared BP in scoliosis and non-scoliosis subjects. According to the data reported in our review, scoliosis presents with asymmetrical pain, which is, for most of the time, lumbar and at the curve’s apex, eventually radiating to one leg. Radiating symptoms are usually localised on the front side of the thigh (cruralgia), while sciatica is more frequent in non-scoliosis subjects. These radiating symptoms relate to rotational olisthesis [6,13], consistently with other reports [20,21]. Also, the type and localization of the curve have an impact, with lumbar and thoracolumbar ones being more painful than thoracic [7]. In thoracic curves, the painful area is usually distal to the curve [7].

Other features of pain in scoliosis are related to difficulty standing and eventually sitting for a prolonged time, where lying down seems to relieve symptoms [5]. Travelling and lifting seem challenging for patients with curves between 20° and 40°, while for those with larger curves, walking seems problematic [5]. We can hypothesise that these symptoms are associated with spine stiffness, which typically characterises scoliosis, and the altered biomechanics of the spine due to frontal and/or sagittal imbalance. We can also speculate that upper spine pain and fatigue are symptoms that start earlier, before degeneration, and could be more related to the altered biomechanics of the spine, whereas radiating LBP is a typical complaint of patients with degeneration in the lumbar spine; however, more clinically descriptive studies are needed to investigate these speculations.

Data from the papers included in this review are inconsistent regarding pain intensity and the duration of symptoms. Some studies reported more intensity and duration of symptoms in adults with scoliosis and BP [5,7]. In contrast, others found no difference compared to the control groups of non-scoliosis subjects [16,17].

Reporting about disability is challenging, too. Data collected from the ODI show no differences between scoliosis and non-scoliosis subjects [16]; however, some differences appear with the Roland Morris Scale and the McGill Questionnaire [5]. The ODI may not be suitable for capturing the disability of scoliosis patients. Recently, a study about bracing in adults with scoliosis and BP reported good results on pain and the Core Outcome Measurement Index (COMI), but no changes were recorded for the ODI [22,23]. Therefore, the application of the ODI in this specific group of patients seems questionable, and more specific tools are under investigation and applied in routine clinical practice [24,25].

Scoliosis is a three-dimensional trunk deformity that leads to global imbalance. In adult scoliosis, trunk imbalance has been suggested as one of the most crucial elements in pain generation; however, the studies that suggest this fell outside of the inclusion criteria of our study, mostly because they lacked control groups. The Schwab classification tried to help understand the pattern and risk factors of pain [9]; however, some papers questioned the role of such parameters in lumbar degenerative scoliosis [26]. As the evidence grows, we hope that the quality of evidence is such that we can compare the role of trunk imbalance in scoliosis and non-scoliosis populations and the relationship to LBP. As we already stated, it is possible that a patient with scoliosis experienced nonspecific LBP, and the findings of this review will help clinicians in everyday practice. It is important to recognise specific features of pain to correctly classify patients with scoliosis and BP to provide appropriate specific treatment. We need to bear in mind that LBP is very frequent in the adult population, and the disadvantaged biomechanics of the spine with scoliosis can represent a risk factor for these patients. If the features of pain are well-known, specific treatment can be applied when appropriate, with exercise [27] and bracing showing different degrees of effectiveness [28].

Due to the increasing prevalence of spinal deformities in adulthood, linked to the progressive ageing of the population, and the need for clinicians to identify a clear clinical picture for appropriate treatments, it is of major importance to identify what is known (signposting the relevant papers to clinicians) and what is unknown (driving future research efforts). A systematic review is an appropriate methodology to answer these needs. Due to the expected scarcity of papers, we considered a wide approach to collect all possible information. What we found clearly shows the need for much more and higher quality research in the field. Clinicians need to know if their patient’s BP is due to a spinal deformity or if it is a common BP similar to patients without deformities. The next research step can be gathering consensus among experts to determine the current clinical understanding and develop research hypotheses for future studies.

### Study Limitations

One limitation was the different outcome measures used in the different studies. A standard method for measuring pain was missing. Some papers applied ordinal scales, and others the NRS. For pain frequency and duration, some reported the year, and others used descriptive scales. All these elements, together with the small number of retrieved studies, prevented performing a meta-analysis. Some adults with scoliosis seek a clinical visit to check the evolution of their curves, while other times, for disability or pain. They may be used to experiencing some pain and fatigue in their everyday life, and therefore it is possible that they are frequently not concerned about their symptoms but may be worried about progression. This behaviour may justify the confusion regarding the pain’s features and characterization. This highlights the need for further studies describing the pain features in scoliosis adults compared to adults with BP without scoliosis.

No study reported a direct comparison of pain in degenerative and idiopathic scoliosis, making it impossible to determine any difference between the two populations. Degenerative de novo scoliosis is not easy to diagnose, and it is possible that clinicians are not sure if it is a de novo scoliosis rather than idiopathic with a delayed diagnosis.

Unfortunately, the quality of the included studies is low. Moreover, just a few of them focused on the clinical features of LBP in adults with scoliosis. More research is needed in the field; therefore, we suggest starting with a consensus among experts to better define the most relevant features to investigate according to the available data and clinical experience and then designing appropriate clinical studies.

## 5. Conclusions

Pain in adults with scoliosis and BP seems to present specific features. Its localization, usually asymmetrical and associated with cruralgia, was the most specific feature. It remains unclear whether pain intensity and duration can differentiate scoliosis and non-scoliosis adults with BP. Further studies are needed to better understand BP in adults with scoliosis and provide specific treatment recommendations.

## Figures and Tables

**Table 1:** Characteristics of the included studies.

Authors	Design	Setting		Evaluated Pain	Participants	Sample		Age		Type of Scoliosis (AAIS or De Novo)	Lumbar Curve Severity in Degrees (Before Treatment)	Presence of More than One Curve
		Recruitment from hospital/outpatient/general population	Country	Low back pain (LBP); Back pain (BP)		N° scoliosis; Female (F) %;	N° non- scoliosis; Female (F)	Scoliosis Mean (SD), Range in years (as reported by study)	Non- Scoliosis Mean (SD), Range in years (as reported by study)	As reported by study	As reported by study	As reported by study
Perennou 1994 [13]	prospective controlled	Spine Rehabilitation Unit	France	LBP	671	50 (7.5%); F 36 (72%)	621 (92.5%); F 298 (48%)	62.1 ± 12.4 (5 pz < 45 y)	49.6 ± 15.5	14% AIS, 86% discovered during examination	21.2 ± 11.4° (56% <20°, 28% 20–29; 10% 30–39; 6% ≥40°	only lumbar curves
Gremeaux 2008 [6]	prospective controlled	Spine Rehabilitation Unit	France	LBP	100	50 (50%) F 68%	50 (50%); F 66%	62 ± 13.1	62 ± 13.7	idiopathic and degenerative scoliosis	23.1 ± 13.1° (10–75°)	only lumbar curves
Yuan 2019 [14]	cross- sectional	Department of Physical Therapy and Rehabilitation	China	LBP	90	41 (45.5%) F 100%	49 (54.5%); F 100%	24.95 ± 2.90	24.73 ± 2.83	100% AIS	26°	only lumbar curves
Jackson 1983 [7]	retrospective controlled	Department of Orthopaedics	Canada	BP	377–245 report pain	197 pts (52%)–101 pts (51%) report pain;	180 pts (48%)–144 pts (80%) report pain	31	36	idiopathic scoliosis	16 pts lumbar 38°	45 pts thoracic curve 60°; 26 pts thoracolumbar 50°; 14 pts double curve 55°;
Mayo 1994 [5]	retrospective cohort study	Departments of Medicine and Department of Occupational Health, and Epidemiology and Biostatistics	Canada/USA	BP	3231 (724 report pain)	1476 (45.6%)–295 pts report pain;	1755 (55.4%)–429 report pain			100% AIS		
Hoevenars 2022 [15]	retrospective controlled	Outpatient	Netherlands	LBP	320	80 (25%); F 79%	240 (75%); F 79%	50.9 (SD 14.1, min–max 21–76)	50.1 (±12.0, 21–74)	24 adult idiopathic scoliosis, 56 de novo degenerative lumbar scoliosis;	21.4 (9.4, 11–72)	only lumbar curves
Bissolotti 2013 [16]	cross- sectional	Outpatient	Italy	LBP	80	40 (50%); F 75%	40 (50%); F 77.5%	61.8 ± 11.5	58.2 ± 10.9	Adult scoliosis	27.1 ± 11.5° primary curve (range, 15–63°); thoracic curve 25.5 ± 22.3° (range, 8–58°)	
Weinstein 2003 [17]	prospective controlled	Department of Orthopaedic Surgery	USA	BP	179 (88 report pain)	117 (65.3)–71 (60%) pts report pain; F 89%	62 (34.6%)–17 (10.4%) pts report pain; F 79.4%	66 (range, 54–80 y)	<65 y: 23/62 (37); >65 y 39/62 (63)	Late-onset idiopathic scoliosis	49.41 (SD 26.38) (range 15–90) lumbar, 89.54 (32.69) (range 50–155) Thoracolumbar, 84.50 (30.17) (range 23–156) thoracic	48 (41%) thoracic curve, 14 (12%) thoracolumbar, 32 (27%) lumbar, 23 (20%) double major

**Table 2:** Critical appraisal of the included studies.

Cross-Sectional	1	2	3	4	5	6	7				
Bissolotti 2013 [16] *	Unclear	No	Yes	No	No	Yes	Unclear				
Yuan 2019 [14]	Yes	Yes	Yes	No	No	Yes	Yes				
**Longitudinal**	**1**	**2**	**3**	**4**	**5**	**6**	**7**	**8**	**9**	**10**	**11**
Gremeaux 2008 [6]	No	Yes	Yes	No	No	No	Yes	Yes	No	No	Yes
Hoevenars 2022 [15]	Yes	Yes	Yes	No	No	No	Yes	Yes	No	No	Yes
Jackson 1983 [7]	Yes	Yes	Yes	No	No	No	Yes	Unclear	Yes	Unclear	Unclear
Mayo 1994 [5]	No	Yes	Yes	Yes	Yes	No	Yes	Yes	No	No	Yes
Perennou 1994 [13]	No	Yes	Yes	No	No	No	Yes	Unclear	Unclear	Unclear	Yes
Weinstein 2003 [17]	No	Yes	Yes	No	No	No	Yes	Yes	No	No	Yes

**Cross-sectional studies items**: (1) Were the criteria for inclusion in the sample clearly defined? (2) Were the study subjects and setting described in detail? (3) Were objective, standard criteria used for the measurement of the condition? (4) Were confounding factors identified? (5) Were strategies to deal with confounding factors stated? (6) Were the outcomes measured in a valid and reliable way? (7) Was appropriate statistical analysis used? **Longitudinal studies items**: (1) Were the two groups similar and recruited from the same population? (2) Were the exposures measured similarly to assign people to both exposed and unexposed groups? (3) Was the exposure measured in a valid and reliable way? (4) Were the confounding factors identified? (5) Were strategies to deal with the confounding factors stated? 6) Were the groups/participants free of the outcome at the start of the study (or at the moment of exposure)? (7) Were the outcomes measured in a valid and reliable way? (8) Was the follow-up time reported and was it sufficient to be long enough for outcomes to occur? (9) Was a follow-up complete, and if not, were the reasons for the lack of a follow-up described and explored? (10) Were strategies to address incomplete follow-up utilised? (11) Were the strategies to address the incomplete follow-up utilised? * Conference abstract.

**Table 3:** Symptom characteristics provided by the included studies.

Authors	Severity/Intensity of Pain		Location of BP		Referred/Lower Extremity Symptoms		Functional Status	
	Scoliosis	Non-Scoliosis	Scoliosis	Non-Scoliosis	Scoliosis	Non-Scoliosis	Scoliosis	Non-Scoliosis
Perennou 1994 [13]	_	_	_	_	40% radicular pain: 26% Sciatica, 14% cruralgia	44.3% radicular pain: 38% Sciatica, 6.3% cruralgia	_	_
Gremeaux 2008 [6]	60% little or usual; 40% considerable or severe	68% little or usual, 32% considerable or severe	_	_	56% (sciatica 26%; cruralgia 26%, neurological claudication 10%, buttock pain 30%), Inguinal dysesthesia 30%, 10% costo-iliac syndrome; Buttock pain (20% little or usual; 45% considerable or severe) Inguinal pain (16.6%; 70%) Obturator neuralgia (3.3%; 30%)	44% (sciatica 32%; cruralgia 12%, neurological claudication 8%, buttock pain 34%) Inguinal dysesthesia 6%; 0% costo-iliac syndrome	_	_
Yuan 2019 [14]	3.5 NRS	5.5 NPRS	32 (78%) left-sided lumbar pain, 9 (21%) right-sided lumbar pain; 78% pain on the convex side	83.7% midline or symmetrical pain		_	_	_
Jackson 1983 [7]	3.3 (scale from 0 to 5)	_	44% pain at lower junctional segment /compensatory curves below the major deformity; DM: 35% mainly junctional area, 44% localised pain in lower junctional levels and in lesser curves below. TL and L mainly junctional and fractional curve segments below MC in 46% and 44%; lumbosacral half-curve segment was most painful.		65% of patients complained of limb distress, including buttock and thigh pain, before treatment.	_	_	_
Mayo 1994 [5]	_	_	Spreading pain (curves > 40°), generalised back pain (curves > 20°)	_	_	_	Limitations in lifting, walking, standing, travel, sitting. Need to change position and lie down/rest.	_
Hoevenaars 2022 [15]	58.4 (19.1) NRS	60.4(19.1)	_	_	_	_	39.5 (±12) ODI	40.2 (±12.1) ODI
Bissolotti 2013 [16]	NRS 5.9 ± 1.8 (range 2–10)	5.1 ± 2.2	_	_	27% sciatic pain	47% (sciatic pain)	33.9 ± 17% ODI	32.6 ± 18.8% ODI
Weinstein 2003 [17]	Little/moderate score 1–2: 48/71 (68%); quite bad/unbearable score 3–5: 23/71 (32%)	Little/moderate score 1–2: 12/17 (71%); quite bad/unbearable score 3–5: 5/17 (29%)	_	_	_	_	37 pts (39%) felt they had a disability	16 pts (30%) felt they had a disability

Abbreviations: pts: patients; BP: back pain; DM: double major; TL: thoracolumbar; L: lumbar; MC: major curve.

## Data Availability

Not applicable.

## Appendix A

Inception to May 2023

Databases: PubMed (via pubmed-ncbi.nlm.nih.gov/ accessed on 15 May 2023), CINHAL (via EBSCOhost), EMBASE (via Embase.com) and Scopus, from inception to May 2023.

PubMed (via pubmed-ncbi.nlm.nih.gov/ accessed on 15 May 2023)

1. (“spinal curvatures”[MeSH Terms]) OR (scoliosis[MeSH Terms]);
2. ((“spinal curvatures*”[Title/Abstract]) OR (scoliosis*[Title/Abstract]) OR (“spinal deformit*”[Title/Abstract]);
3. #1 OR #2;
4. (back pain[MeSH Terms] OR sciatica[MeSH Terms] OR radiculopathy[MeSH Terms]);
5. ((low back pain*[Title/Abstract]) OR (back pain*[Title/Abstract]) OR (spinal pain[Title/Abstract]) OR (backache*[Title/Abstract]) OR (back ache*[Title/Abstract]) OR (aching[Title/Abstract]) OR (lumbar pain[Title/Abstract]) OR (lumbo*[Title/Abstract]) OR (back disorder*[Title/Abstract]) OR sciatic*[Title/Abstract] OR radiculopat*[Title/Abstract]);
6. #4 OR #5;
7. #3 AND #6.

EMBASE (via Embase.com)

8. (‘scoliosis’/exp OR ‘spinal pain’/exp OR ‘spine malformation’/exp);
9. (‘spine diseas*’:ab,ti,kw OR ‘spinal curvature*’:ti,ab,kw OR ‘idiopathic* scoliosis’:ti,ab,kw OR ‘degenerative* scoliosis’:ti,ab,kw OR ‘de novo* scoliosis’:ti,ab,kw OR ‘spine malformat*’:ti,ab,kw OR ‘spinal deformit*’:ti,ab,kw OR ‘scoliosis*’:ti,ab,kw);
10. #1 OR #2;
11. ‘backache’/exp OR ‘sciatica’/exp;
12. (‘backache*’:ti,ab,kw OR ‘back pain*’:ti,ab,kw OR ‘low back pain*’:ti,ab,kw OR ‘scoliosis*’:ti,ab,kw OR ‘spinal pain*’:ti,ab,kw OR ‘back ache*’:ti,ab,kw OR ‘lumbar pain*’:ti,ab,kw OR ‘lumbo*’:ti,ab,kw OR ‘aching’:ti,ab,kw OR ‘back disorder*’:ti,ab,kw OR ‘sciatic*’:ti,ab,kw OR ‘radiculopat*’:ti,ab,kw);
13. #4 OR #5;
14. #3 AND #6.

Scopus

15. TITLE-ABS-KEY(“spinal curvature*” OR “scoliosis*” OR ((“idiopathic*” OR “degenerativ*” OR “de novo*”) W/1 (“scoliosis”)));
16. TITLE-ABS-KEY(“back pain*” OR “low back pain*” OR ((“spinal” OR “lumbar”) W/1 (“pain*”)) OR “backache*” OR “back ache*” OR “aching” OR “lumbo*” OR “back disorder*” OR “sciatic*” OR “radiculopat*”)));
17. #1 AND #2.

CINAHL (via EBSCOhost)

18. (MH “Spinal Curvatures+”) OR (MH “Scoliosis+”);
19. TI ((spinal W1 curvatures*) OR “scoliosis*” OR ((idiopathic* OR degenerativ* OR de novo*) N1 (scoliosis))) OR AB ((spinal W1 curvatures*) OR “scoliosis*” OR ((idiopathic* OR degenerativ* OR de novo*) N1 (scoliosis))) OR SU ((spinal W1 curvatures*) OR “scoliosis*” OR ((idiopathic* OR degenerativ* OR de novo*) N1 (scoliosis)));
20. #1 OR #2;
21. (MH “Back Pain+”) OR (MH “Sciatica”) OR (MH “Radiculopathy”);
22. TI (((back OR spinal OR lumbar) N1 (pain*)) OR backache OR sciatic* OR radiculopat*OR (back W1 ache*) OR aching OR lumbo* OR (back W1 disorder*)) OR AB (((back OR spinal OR lumbar) N1 (pain*)) OR backache OR sciatic* OR radiculopat*OR (back W1 ache*) OR aching OR lumbo* OR (back W1 disorder*)) OR SU (((back OR spinal OR lumbar) N1 (pain*)) OR backache OR sciatic* OR radiculopat*OR (back W1 ache*) OR aching OR lumbo* OR (back W1 disorder*));
23. #4 OR #5;
24. #3 AND #6.
